# Longitudinal Assessment of Global and Regional Rate of Grey Matter Atrophy in 1,172 Healthy Older Adults: Modulation by Sex and Age

**DOI:** 10.1371/journal.pone.0114478

**Published:** 2014-12-03

**Authors:** Fabrice Crivello, Nathalie Tzourio-Mazoyer, Christophe Tzourio, Bernard Mazoyer

**Affiliations:** 1 Université de Bordeaux, GIN, UMR 5296, Bordeaux, France; 2 CNRS, GIN, UMR 5296, Bordeaux, France; 3 CEA, GIN, UMR 5296, Bordeaux, France; 4 INSERM, U708, Victor Segalen University, Bordeaux, France; University Of Cambridge, United Kingdom

## Abstract

To characterize the neuroanatomical changes in healthy older adults is important to differentiate pathological from normal brain structural aging. The present study investigated the annualized rate of GM atrophy in a large sample of older participants, focusing on the hippocampus, and searching for modulation by age and sex. In this 4-year longitudinal community cohort study, we used a VBM analysis to estimate the annualized rate of GM loss, at both the global and regional levels, in 1,172 healthy older adults (65–82 years) scanned at 1.5T. The global annualized rate of GM was −4.0 cm^3^/year (−0.83%/year). The highest rates of regional GM loss were found in the frontal and parietal cortices, middle occipital gyri, temporal cortex and hippocampus. The rate of GM atrophy was higher in women (−4.7 cm^3^/year, −0.91%/year) than men (−3.3 cm^3^/year, −0.65%/year). The global annualized rate of GM atrophy remained constant throughout the age range of the cohort, in both sexes. This pattern was replicated at the regional level, with the exception of the hippocampi, which showed a rate of GM atrophy that accelerated with age (2.8%/year per year of age) similarly for men and women. The present study reports a global and regional description of the annualized rate of grey matter loss and its evolution after the age of 65. Our results suggest greater anatomical vulnerability of women in late life and highlight a specific vulnerability of the hippocampus to the aging processes after 65 years of age.

## Introduction

As life expectancy continues to increase, dementia prevalence rises, thus prompting considerable interest in characterizing neuroanatomical changes in healthy older adults for the purpose of differentiating between pathological and normal brain aging [1]–[5]. While it exits a rich history of structural brain aging, few cross-sectional magnetic resonance imaging (MRI) studies have been performed in very large samples older adults derived from the general population. These studies have demonstrated both global and regional grey matter (GM) volume and/or cortical thickness decline during aging [6]–[8]. However, for quantification of cerebral aging, tissue atrophy rate may be a more sensitive marker than single time-point measurements of atrophy [5], [9]–[13]. In particular, it is apparently important to ascertain whether in older adults the speed of atrophy remains constant or accelerates, globally and regionally [4], [14], and longitudinal studies, rather than cross-sectional studies, are required to enable individual and accurate measurements of the speed of GM change. However, longitudinal studies of global and regional brain atrophy in large cohorts of healthy older adults participants remain lacking. This is mainly due to the high cost and complex organizational structure of large population-based studies, especially those dealing with a narrow age range. Furthermore, few studies have investigated the impacts of aging risk factors and determinants (e.g., age, sex, or hypertension) on such GM atrophy rates, together with the pattern of age-related change (i.e., age trajectories of cortical atrophy) among general populations of older adults.

Resnick et al. found no differences according to sex in their investigation of longitudinally estimated rates of GM change among 24 participants (upon 92 non-demented individuals) who remained very healthy during the 4-year follow-up (11 males; mean age: 69.5 years, range: 59–85 years) [15]. In contrast, Thambisetty et al. studied 66 older adults (38 males; mean age: 68 years, range: 60–84 years) and found that compared to women, men showed a greater rate of cortical thickness decline in the middle frontal, inferior and superior parietal, parahippocampus, poscentral, and superior temporal regions [16]. To our knowledge, these two investigations of relatively small study samples are the only ones that have investigated the effect of sex on the GM atrophy rate in older adults. Additionally, two non-longitudinal cross-sectional studies were performed in large samples of the general elderly population; they found no significant effect of sex on GM volume atrophy, but observed a tendency for greater atrophy in women [6] (*n* = 662; 331 men; mean age: 69 years) or a higher atrophy of the frontal lobe cortical thickness in men [8] (*n* = 1,022; 488 men; mean age: 68 years). These results do not resolve the question of the effect of sex on GM change.

Fotenos et al. investigated the acceleration of cerebral atrophy with age (i.e., the effect of age on the longitudinal rate of tissue change) in 94 healthy participants over 65 years of age, and found that the rate of cerebrum atrophy did not accelerate with age [17]. However, pooling GM and WM makes it impossible to evaluate age-related changes of the rate of GM atrophy alone. To date, very few studies have investigated the effect of age trajectories on local GM atrophy in older adults. Previous studies have mainly focused on target regions of interest, especially the hippocampus [14], [18]. Among these, Fjell et al. reported significant associations between age and rate of GM cortical thickness change—principally found in the entorhinal cortex and the hippocampus, and also observed in the isthmus of the cingulate, middle temporal, and parahippocampal gyri [4]. The scarcity of studies investigating this subject, together with the small study sample size, does not allow any certain conclusion to be drawn regarding whether the age-related increase of regional atrophy reflects a pattern of late aging in healthy older adults, or reflects preclinical symptoms of dementia [13]. Moreover, to our knowledge, no study has yet investigated the interaction between age and sex in relation to the rate of GM atrophy at the global and regional levels in older adults, leaving open the issue of sex modulation in acceleration of the rate of GM change in late adult life.

The present study addressed these debated issues, taking advantage of the on-going longitudinal “Three Cities” (3C) study in which 1,172 non-demented older adults (age ≥65 years) underwent brain MRI scanning twice with a 4-year interval. Using validated and automated MRI analysis, we investigated the global and regional patterns of the annualized rate of GM atrophy, focusing on the hippocampus, and searching for modulation by age and sex.

## Methods

### 1. Participants

The 3C Study is an on-going population-based prospective investigation of the relationship between vascular risk factors and dementia [19]. It is being carried out in three French cities: Bordeaux (southwest), Montpellier (southeast), and Dijon (central east). A sample of non-institutionalized participants aged 65 years and over was randomly selected from the electoral rolls of each city. Between January 1999 and March 2001, 9,686 participants fulfilling the inclusion criteria agreed to participate. The ethics committee of the Kremlin-Bicêtre hospital approved the 3C protocol, and all participants signed an informed consent. Following recruitment, 392 participants withdrew from the study, leaving 9,294 participants (2,104 in Bordeaux, 4,931 in Dijon, and 2,259 in Montpellier). In the 4,931 participants of the Dijon city, a cerebral MRI examination was proposed to those aged 65–80 years who were enrolled between June 1999 and September 2000 (*n* = 2,763). Although 2,285 subjects (82.7%) agreed to participate, only 1,924 MRI examinations were performed due to financial limitations. Approximately 4 years after inclusion, 1,402 of these participants agreed to have a follow-up MRI (follow-up rate: 77.8%), of whom 230 were later excluded due to poor technical quality of the anatomical images, failure in MRI processing, or missing data (demographic, biological, cognitive, genetic status, or previous history of stroke or diagnosis of dementia). The final 3C-MRI longitudinal study sample (including only individuals from Dijon) comprised 1,172 participants of 65–82 years of age, including 430 men and 742 women.

### 2. Participants' cognitive and health status

The education level of each participant (measured in number of school years starting from primary school) was recorded at baseline. Baseline global cognitive status was determined using the Mini-Mental State Examination (MMSE) [20]. Dementia diagnosis and classification were made by the 3C Study local investigators according to the criteria of the Diagnostic and Statistical Manual of Mental Disorders-IV (American Psychiatric Association, 1994), and were validated by a panel of independent neurologists. According to this diagnosis, participants with incident dementia were excluded.

At baseline, participants were subjected to fasted blood sampling, and standard biological parameters were measured, including plasma cholesterol and blood glucose level. Participants were considered hypertensive if their systolic blood pressure was>140 mmHg, diastolic blood pressure was>90 mmHg, or if they were on antihypertensive medications at study entry. Smoking status was categorized as either current smoker or nonsmoker (including former smokers). Participants' depressive symptoms were assessed with the Center for Epidemiological Studies-Depression (CES-D) score. Finally, women were asked if and for how long they had been taking hormone replacement therapy (HRT).

### 3. MRI acquisition

All baseline and follow-up structural brain scans were acquired using the same MRI machine (1.5 T; Siemens, Erlangen) and the same standardized image acquisition protocol. The conventional exclusion criteria were applied: carrying a cardiac pacemaker, valvular prosthesis, or other internal electrical/magnetic device; history of neurosurgery or aneurysm; presence of metal fragment in the eyes, brain, or spinal cord; and claustrophobia.

Positioning in the magnet was based on a common landmark for all participants—the orbito-meatal line—so that the entire brain, including cerebellum and mid-brain, was contained within the field of view of acquisition. First, the three-dimensional (3D) high-resolution T1-weighted brain volume was acquired using a 3D inversion recovery fast spoiled-gradient echo sequence (3D SPGR; TR: 9.7 ms; TE: 4 ms; TI: 600 ms; coronal acquisition). The axially reoriented 3D volume matrix size was 256×192×256, with a voxel size of 1.0×0.98×0.98 mm^3^. Second, T2-weighted brain volumes were acquired using the same 2D fast spin-echo sequence with two echo times (TR: 4,400 ms; TE1: 16 ms; TE2: 98 ms). T2 acquisition consisted of 35 3.5-mm-thick axial slices (with 0.5-mm spacing between slices), having a matrix size of 256×256, and an in-plane resolution of 0.98×0.98 mm^2^. T1 and T2 datasets were readily reconstructed, and visually checked for major artifacts before further analysis.

### 4. MRI processing

In a first step we pre-processed the T1 and T2 images. At both baseline and follow-up taken separately, the T2 images were affine only aligned to the T1 images using a 6 parameters transformation (3 rotations and 3 translation) [21]. Then the T1 follow-up image was also rigidly aligned to the T1 baseline image. At this stage, the T1 at baseline, the T2 at baseline and the T1 at follow-up images of an individual were rigidly aligned. The concatenation of the transformation matrices computed to align the T2 follow-up image to the T1 follow-up image with the transformation matrices estimated to align the T1 follow-up to the T1 baseline allows to additionally align the T2 follow-up image to the T1 baseline image, thus providing a set of 4 images per participants aligned to the T1 baseline one.

In a second step, these images were then analyzed with a Voxel-Based morphometry (VBM) protocol [22] using SPM99 normalization and segmentation procedures. To account for the structural characteristics of the aged brain, the VBM protocol was optimized in the following two main ways.

1) The first optimization consisted in the creation of specific template representative of the population studied in terms of demography and signal characteristics (signal to noise ration, resolution and contrast). Grey matter (GM), white matter (WM), and cerebro-spinal fluid (CSF) templates specific to the 3C-MRI cohort were thus created using a sub-sample of 150 men and 150 women. The sample of 150 men (respectively 150 women) was match for age, proportion with high blood pressure, and education level to the entire group of men (respectively women).

Once these templates created, each subject's baseline T1 image was first segmented using the SPM MRI default priors to obtain a GM partition image in its native acquisition space. This GM image was then spatially non-linearly normalized to the specific 3C-MRI priors previously built using the SPM software. A affine registration is followed by nonlinear deformations defined by linear combinations of 8×8×8 3D discrete cosine transform basis functions This results in each of the deformation fields being described by 1,536 parameters representing the deformations in three orthogonal directions. The corresponding deformation fields (i.e. the non-linear spatial normalization parameters) were reapplied to the 4 native images (T1 and T2 at both baseline and follow-up). Resulting T1 normalized volumes were only then segmented using the same 3C-MRI priors—providing GM, WM, and CSF partition images for both baseline and follow-up MRI sessions. For all individuals of the 3C-MRI cohort, visual inspection was performed after both non-linear spatial normalization and tissue segmentation for ensuring optimal GM tissue extraction.

2) The second optimization was dedicated to obtain a good segmentation of the CSF compartment and therefore of the total intracranial volume. Accordingly, we performed for both baseline and follow-up MRI sessions an additional multi-spectral segmentation with both the T1 and T2 volumes, again using the 3C-MRI priors. Two optimized intracranial volume spaces were defined as the sum of the resulting GM, WM, and CSF partition images from each MRI session. Then a common intracranial space, computed, as the intersection of the two intracranial spaces, was defined based on the hypothesis that the total intracranial volume of the older adult participants did not change during the four-year timespan of the study. Finally, baseline and follow-up optimized CSF partition images were obtained by subtracting the sum of the GM and WM compartment images (obtained through the first T1 mono-spectral segmentation) from the common intracranial space image. Note that the improvement provided by this modified CSF segmentation scheme was previously quantified by comparing the absolute CSF volumes obtained with or without inclusion of T2 images in the segmentation process [6].

To summarize, only the final CSF partition images obtained from multi-spectral segmentation were used subsequently while we used the GM and WM partition images derived from the mono-spectral segmentation of only the T1 volumes, thus not introducing partial volume effect in the GM compartment volume estimation due to the lower resolution of the T2 scan.

### 5. Total intracranial, GM, and hippocampal volume estimations

We applied a modulation step to each individual tissue map in order to preserve the subject's original tissue quantity after being transferred to the reference space used [22]. This involved multiplying (or modulating) voxel values in the segmented images by the Jacobian determinants derived from the spatial normalization step. As a consequence, analysis of modulated data tested for regional differences in the absolute amount (volume) of GM. At baseline and follow-up, GM, WM, and CSF volumes were thus estimated as the integral of voxel intensities over the sum of the corresponding modulated tissue partition image and total intracranial volume (TIV). Hippocampal volume was automatically calculated by integrating the voxel intensities of the modulated GM partition images within hippocampal limits derived from a model of macroscopic neuroanatomical parcellation [23]. This parcellation, the Automated Anatomical Labellng atlas (AAL) [24], was based on the high-resolution single-subject T1 volume provided by the Montreal Neurological Institute.

### 6. Longitudinal follow-up of GM changes

The individual annual rate of GM volume change, expressed in cm^3^/year, was defined as ΔGM_Volume_  =  (GM_Follow-up_ − GM_Baseline_)/(t_Follow-up_ − t_Baseline_), where GM_Baseline_ and GM_Follow-up_ are the estimated GM volumes at baseline and 4-year follow-up, respectively, and (t_Follow-up_ − t_Baseline_) is the individual delay between the two MRI scans. Similarly, the individual annual rate of hippocampus volume change, expressed in cm^3^/year, was defined as ΔH_Volume_  =  (H_Follow-up_ − H_Baseline_)/(t_Follow-up_ − t_Baseline_), where H_Baseline_ and H_Follow-up_ are the estimated hippocampal volumes at baseline and 4-year follow-up, respectively. Finally, the normalized, segmented, modulated images were smoothed with a 12-mm^3^ full-width at half-maximum Gaussian kernel, and GM pairwise difference maps were computed as the difference between follow-up and baseline GM probability maps, divided by the delay between the two MRI scans.

### 7. Statistical analysis

Statistical analyses of volumes and rates of change were performed using JMP9.0.0 (© 2010 SAS Institute, Inc.). A full factorial ANCOVA was used to assess the effects of sex, age, and their interaction on rates of GM and hippocampus atrophy—with educational level, hypertension and TIV included as covariates. To test the internal consistency of our results, we first used the previous full-factorial ANCOVA on the GM and hippocampus volumes measured at baseline. Second, we added to this model a quadratic age effect component (β_0_+β_1_Age+β_2_Age^2^+…+*ε*) to test the potential non-linear pattern of age-related change on these volumes. To test for a potential effect of HRT on atrophy, we compared the rates of GM atrophy between a sample of 325 women who did not receive HRT at menopause (HRT−) and a sample of 166 women who did receive HRT (HRT+). These two samples were matched for age, proportion of hypertensive participants, and educational level.

Statistical analyses of voxelwise rate-of-GM-atrophy maps were performed using the SPM5 software package for which the significance level was set at *p*<0.05, with family-wise error (FWE) corrected for multiple comparisons. A full-factorial ANCOVA was used to assess the effects of sex, age, and their interaction on the voxelwise rate-of-GM-atrophy maps, including educational level, hypertension and TIV as covariates.

To ensure that noise during the regression will not cause an attenuation effect of the regression slope, which may lead to a smaller value than the actual slope, we estimated the cross-sectional atrophy rate with a shifting windows approach. The average GM volume of 1-year windows for all the 17 years of the study was computed and the average volume difference between adjacent age windows calculated.

In order to provide additional information on the regional pattern of the annualized rate of GM atrophy (estimate of effect size and variance), we computed, using the full factorial ANCOVA previously described, this rate within regions of interest (summing the left and right hemisphere) defined by the AAL atlas (Tzourio-Mazoyer et al., 2002).

## Results

### 1. Characteristics of the 3C-MRI sample

Table 1 summarizes the characteristics of the 3C-MRI sample used in the present study (*n* = 1,172) as compared to the remainder of the 3C Dijon cohort (*n* = 3,759). On average, the 3C-MRI participants were younger (72.2 vs. 75.5 years; *p*<0.001), and exhibited better health and cognitive status, with a higher level of education (9.8 vs. 9.0 years; *p*<0.001), coupled with a higher MMSE score (27.9 [24]–[30] vs. 27.0 [7]–[30]; *p*<0.001). The 3C-MRI sample also presented a smaller proportion of hypertensive participants (74.7% vs. 81.6%; *p*<0.001), a lower CES-D score (9.9 vs. 11.2; *p*<0.001), and a smaller fraction of participants with diabetes mellitus (5.0% vs. 5.2%; *p*<0.001).

**Table 1:** 3C-Dijon cohort characteristics.

	No MRI	MRI	*p* value
Number of participants (N)	3,759	1,172	-
Age in years	75.5 (5.9	72.2 (3.9	<0.001†
	(65–100)	(65–82)	
Percent men	38.8	36.7	0.22‡
Percent right-handers	92.9	92.4	0.78‡
Years of education	9.0 (4.2)	9.8 (4.3)	<0.001†
	(0–17)	(0–17)	
MMSE score	27.0 (2.3)	27.9 (1.5)	<0.001†
	(7–30)	(24–30)	
Percent with hypertension	81.6	74.7	<0.001‡
Percent current smokers	5.0	5.8	0.19‡
CES-D score	11.2 (9.2)	9.9 (8.7)	<0.001†
	(0–51)	(0–52)	
Cholesterolemia in mmol/L	5.83 (1.00)	5.77 (0.94)	0.06†
	(2.36–10.63)	(3.34–10.73)	
Fasting plasma glucose in mmol/L	5.2 (1.2)	5.0 (1.0)	<0.001†
	(3.1–22.4)	(3.7–17)	

Values are presented as mean (standard deviation) (range).

MMSE: Mini-Mental State Examination.

† Student's *t*-test.

‡ Pearson's chi-squared test.

Table 2 presents the characteristics of the study sample at the time of the first MRI exam, according to sex. Compared to women, men had a higher level of education (10.7 vs. 9.2; *p*<0.001). MMSE scores did not differ between sexes. Compared to women, men presented larger proportions of hypertensive participants (83.8% vs. 69.5%; *p*<0.001), current smokers (9.5 vs. 3.6; *p*<0.001), and participants with diabetes mellitus (5.2% vs. 4.9%; *p*<0.001). Compared to men, women were found to have higher CES-D scores (11.5 vs. 7.1; *p*<0.001) and a higher prevalence of hypercholesterolemia (5.89 vs. 5.54; *p*<0.001).

**Table 2:** 3C-MRI sample characteristics at study entry according to sex.

	Men	Women	*p* value
Number of participants	430	742	-
Age in years	72.0 (4.0)	72.3 (3.9)	0.17†
	(65–82)	(65–81)	
Percent right-handers	91.6	92.8	0.62‡
Years of education	10.7 (4.6)	9.2 (4.0)	<0.001†
	(0–17)	(0–17)	
MMSE score	28.0 (1.4)	27.9 (1.6)	0.30†
	(24–30)	(24–30)	
Percent with hypertension	83.8	69.5	<0.001‡
Percent current smokers	9.5	3.6	<0.001‡
CES-D score	7.1 (6.4)	11.5 (9.4)	<0.001†
	(0–41)	(0–52)	
Cholesterolemia in mmol/L	5.54 (0.92)	5.89 (0.93)	<0.001†
	(3.34–8.71)	(3.45–10.73)	
Fasting plasma glucose in mmol/L	5.2 (1.1)	4.9 (1.0)	<0.001†
	(3.7–15.9)	(3.7–17)	

Values are presented as mean (standard deviation) (range).

MMSE: Mini-Mental State Examination.

† Student's *t*-test.

‡ Pearson's chi-squared test.

### 2. Rate of GM atrophy

**Figure 1:**
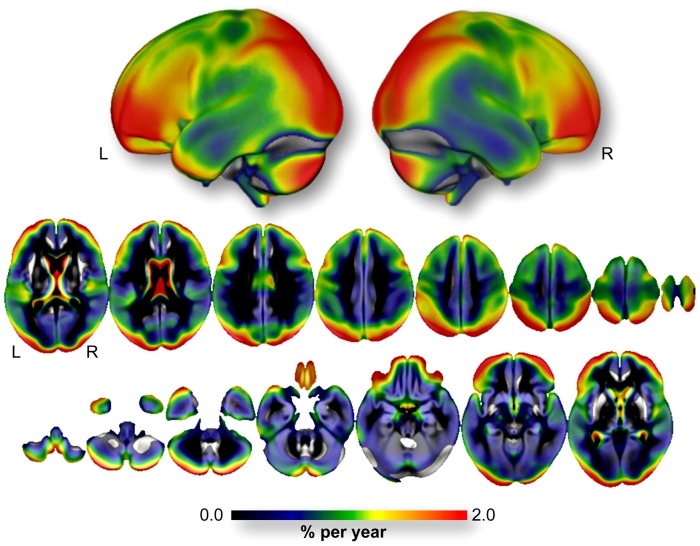
Voxel-wise annualized rate of GM atrophy (expressed in %/year) computed over the entire 3C-MRI sample (*n* = 1,172). Only voxels showing significant annual loss (*p*<0.05 FWE corrected for multiple comparison) are superimposed onto the 3D rendering (*upper part*) and 2D slices of the mean GM map of the 3C-MRI sample. **L**: Left. **R**: Right.

**Figure 2:**
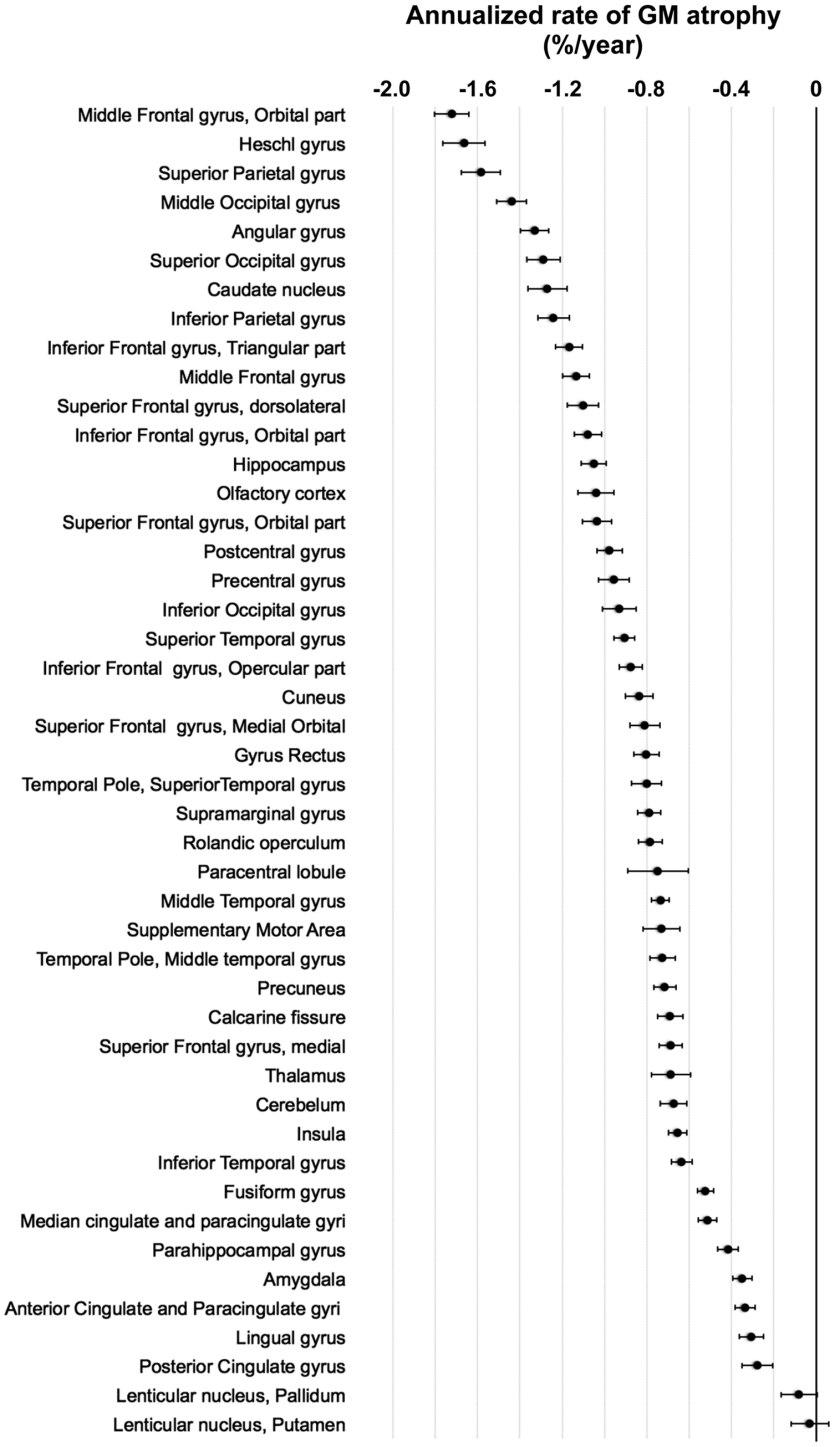
Annualized rate of regional GM atrophy (expressed in %/year) estimated over the entire 3C-MRI sample (*n* = 1,172). Regions of interest were derived from the Automated Anatomical Labelling atlas (AAL) and represent the sum of the left and right hemispheres. Estimated mean and 95% confidence interval of annualized rate of regional GM atrophy are plotted. Annualized rate were normalized by baseline region of interest volumes.

The baseline average GM volume was 509.8±49.9 cm^3^. The annualized rate of GM atrophy was −4.0±3.0 cm^3^/year, representing a GM loss of −0.8%/year. Figure 1 shows the topographic regional distribution of the annualized rate of GM atrophy and illustrates the heterogeneity of the regional rates of GM atrophy. Higher rates were found in the inferior, middle and superior frontal gyri, including their orbital and lateral parts, in the superior and inferior parietal gyri including the angular gyrus and in the middle and superior occipital gyri, in the Heschl gyri and in the hippocampi. To a lesser extent, we also observed high rates of GM atrophy in the postcentral and precentral gyri, the cuneus and the temporal poles. Figure 2 provides an estimation of the annualized rate of GM atrophy (expressed in %/year) within the (Left *plus* Right) regions of interest defined in the AAL atlas. The rates of GM atrophy ranged from −0.03%/year (in the Putamen) to −1.72%/year (in the orbital part of the middle frontal gyri). One can note that almost the entire frontal cortex, the Heschl gyrus and a large part of the occipito-parietal cortices presented annualized rate of GM atrophy larger than the annualized rate of the whole GM atrophy (orbital part of the middle frontal gyrus: −1.72%/year; Heschl gyrus: −1.66%/year; Superior Parietal gyrus: −1.58%/year; middle occipal gyrus: −1.44%/year; angular gyrus: −1.33%/year; superior occipital gyrus: −1.29%/year, caudate nucleus: −1.27%/year; inferior parietal gyrus: −1.24%/year; triangular part of the inferior frontal gyrus: −1.17%/year; middle frontal gyrus: −1.13%/year; dorsolateral of the superior frontal gyrus: −1.10%/year and orbital part of the inferior frontal gyrus: −1.08%/year, see Figure 2).

Corroborating the voxel-wise analysis, the AAL regions of interest analysis presented in Figure 2 reveals that at the exception of the Pallidum and the Putamen, all areas presented a highly significant annualized rate of GM atrophy. Focusing on the hippocampus volume, we estimated a baseline average hippocampus volume of 6.68±0.83 cm^3^. The annualized rate of hippocampus atrophy was −0.07±0.06 cm^3^/year, thus representing a hippocampus volume loss of −1.05%/year.

### 3. Effect of age on rate of GM atrophy

#### 3.1. Global GM

**Figure 3:**
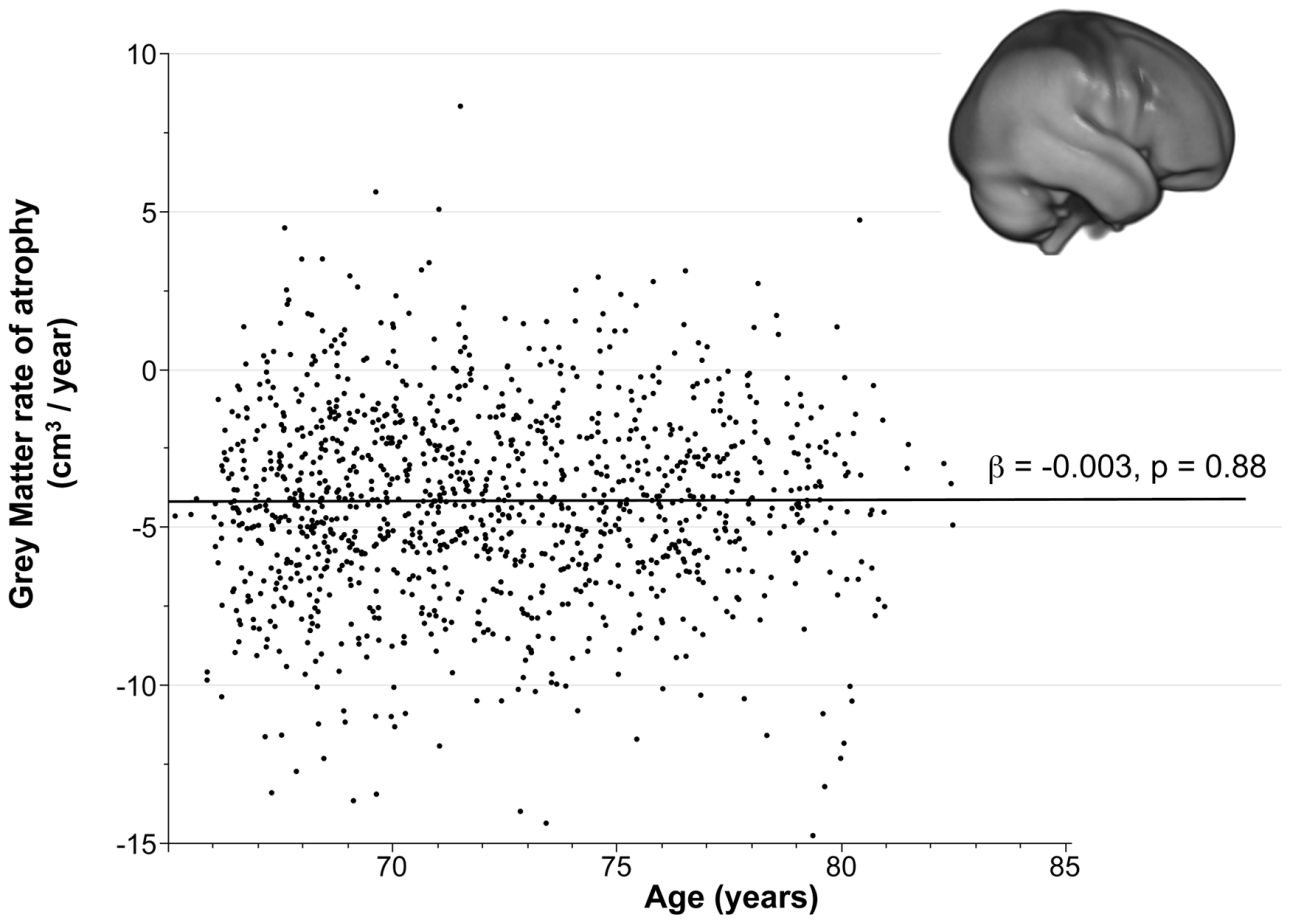
Regression plot of the global annualized rate of GM atrophy vs. subject age, illustrating a steady rate after 65 years of age.

There was no significant age effect on the annualized rate of GM atrophy (*p* = 0.88, Figure 3). The absence of age-related change on the rate of GM atrophy did not significantly differ between men and women according to age-by-sex interaction (*p* = 0.09), indicating that GM loss after 65 years of age occurs at a constant speed (−0.003 cm^3^/year per year of age representing a 0.08%/year increase of the annual GM atrophy rate per year of age) and is independent of sex and age. This absence of age-related change on the annualized rate of GM atrophy was also observed in all GM regions, with the very striking exception of the bilateral hippocampi, where the rate of GM atrophy accelerated with age. This acceleration was independent of sex, as no voxels were statistically found in the age-by-sex interaction.

#### 3.2. Hippocampus

**Figure 4:**
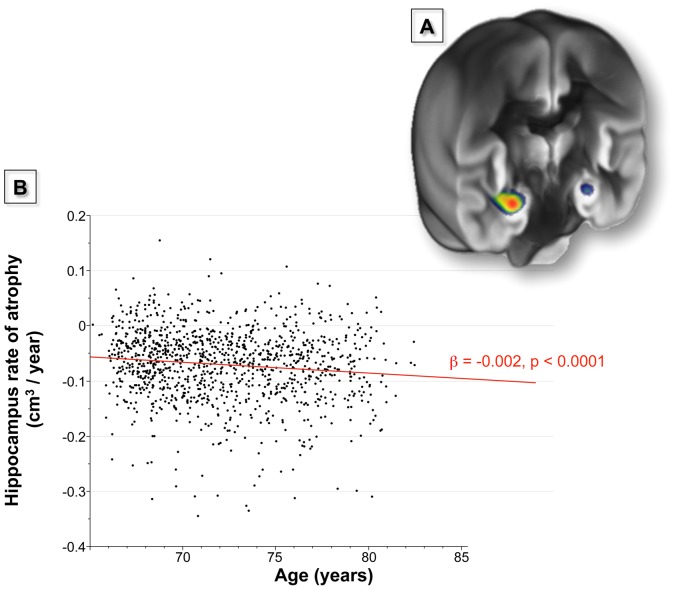
Hippocampal-specific acceleration of the annualized rate of GM atrophy (*p*<0.05 FWE corrected) superimposed on a coronal slice of the mean GM map of the 3C-MRI sample (panel A); Regression plot of the annualized rate of hippocampal GM atrophy vs. subject age (panel B).

We used the hippocampus volume to quantify this acceleration, and found an effect of −0.002 cm^3^/year per year of age (*p*<0.0001), representing a 2.8%/year increase of the annual hippocampus atrophy rate per year of age (Figure 4). Accordingly, the acceleration of the annualized rate of atrophy was 35 times higher in hippocampus than in global GM. Corroborating the voxelwise analysis, this specific local age effect on the hippocampus was found to be equivalent for both sexes (no regional age-by-sex interaction; *p* = 0.83).

### 4. Effect of sex on rate of GM atrophy

#### 4.1. Global GM

**Figure 5:**
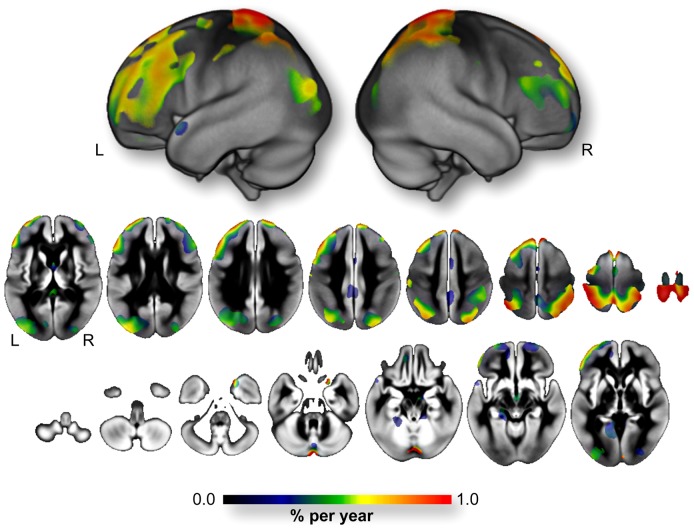
Effect of sex on the voxel-wise annualized rate of GM atrophy (expressed in %/year) illustrating the cortical areas where women presented a higher rate of GM atrophy compared to men. Only voxels showing significant sex-related differences (*p*<0.05 FWE corrected for multiple comparison) are superimposed onto the 3D rendering (*upper part*) and 2D slices of the mean GM map of the 3C-MRI cohort (*n* = 1,172). L: Left. R: Right.

Women exhibited a significantly larger annualized rate of GM atrophy compared to men (−4.7 *vs.* −3.3 cm^3^/year; *p*<0.0001), representing −0.91 and −0.65%/year respectively. Regionally, this difference was mainly observed in the inferior, middle and superior frontal gyri, the poscentral gyrus, the inferior and superior parietal gyri, and the left middle occipital gyrus (Figure 5). By contrast, there was no area in which men showed a higher annualized rate of GM atrophy compared to women.

#### 4.2. Hippocampus

Focusing on the rate of hippocampus atrophy, women presented a higher rate than men, which almost reached significance (−0.075 vs. −0.065 cm^3^/year; *p* = 0.056).

### 5. Effect of HRT on global rate of GM atrophy

Among older adults, HRT+ women presented a significantly lower annualized rate of GM atrophy than HRT− women (−4.1 vs. −4.8 cm^3^/year; *p* = 0.02).

### 6. Effect of education level on global rate of GM atrophy

We found no effect of education level on the rate of GM atrophy (p = 0.63). This absence of association was sex-independent (p = 0.31 for men and p = 0.86 for women).

### 7. Linear versus non-linear models of effects on the age-related changes of GM and hippocampus volumes

#### 7.1. Global GM

Cross-sectional analyses performed at baseline and using the linear model revealed an age-related GM loss of −3.6 cm^3^/year (*p*<0.0001), which was slightly smaller than the effect measured longitudinally (−4.0 cm^3^/year). Adding an (Age)^2^ parameter in the second non-linear model did not improve the fit of the data describing GM tissue loss: the additional quadratic-in-age parameter did not significantly differ from 0 (*p* = 0.22), whereas the strong age effect was still observed and remained of equivalent amplitude with this quadratic fit (−3.6 cm^3^/year; *p*<0.0001). This result is in concordance with the steadiness of the longitudinally measured rate of GM atrophy.

**Figure 6:**
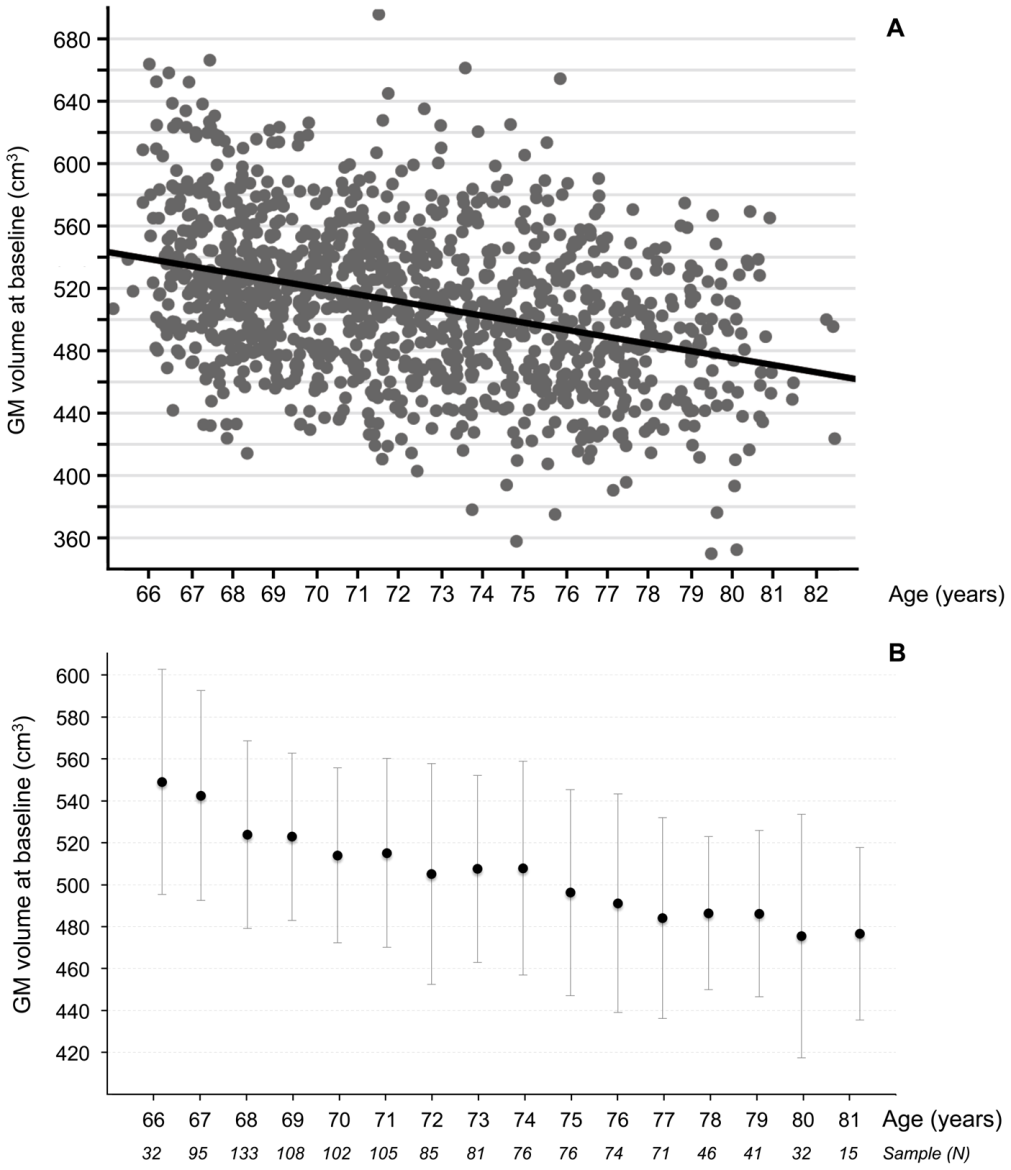
Regression plot of the GM volume at baseline vs. subject age (panel A; average GM volume and standard deviation (bar plot) of one-year windows for all the years of the study (panel B). The sample numbers indicate the number of older participants in each one-year window.

We estimated a loss of −4.53 cm^3^/year (Std Err Mean  = 0.35) for the least square linear regression approach (Figure 6, A part) as compared to −5.04 cm^3^/year (Std Err Mean  = 1.66) with the shifting windows approach (Figure 6, B part). Note that the latest value is not the value predicted by the full factorial ANCOVA and presented just above, but the raw regression slope between GM volume and age of the participants without any covariates. We observed a slightly smaller value using the linear regression reflecting the limited attenuation effect of the regression slope du to the limited noise in the regression. However the differences (Mean and SEM) observe between the two rates are minimal and allow us say that the cross-sectional atrophy rate estimated with the classical linear regression model is not biased.

#### 7.2. Hippocampus

Cross-sectional analyses performed at baseline and using the linear model revealed an age-related hippocampus loss of −0.07 cm^3^/year (*p*<0.0001), which was identical to the longitudinally measured effect (−0.07 cm^3^/year). In contrast to the global GM volume, adding an (Age)^2^ parameter unmasked a significant quadratic-in-age effect on hippocampus volume (*p* = 0.04) revealing a hippocampus atrophy acceleration, in agreement with the aforementioned age effect found on the hippocampus rate of atrophy.

## Discussion

The present study provides a detailed description of the rate of GM atrophy and its evolution after the age of 65, using a longitudinal MRI sample of 1,172 healthy older adult participants. The rates of GM atrophy were found to be highest in the frontal and occipito-parietal cortex, in the hippocampus in the temporal cortex and poles. These rates of GM atrophy appeared to be constant after 65 years, both globally and regionally—with the noticeable exception of the hippocampus where the rate of GM atrophy strongly accelerated with age. Furthermore, we showed that women exhibited higher rates of GM atrophy than men, principally in the frontal and parieto-occipital cortices.

### 1. GM rate of atrophy

#### 1.1. Global GM rate of atrophy

Several previous studies in healthy older adults (aged over 57 years) have reported age-related decreases of GM ranging from −1.4 to −2.6 cm^3^/year [5]–[6], [15], [25]–[28]. These figures are consistent but somewhat smaller than the rate of GM atrophy found in the present longitudinal study (−4.0 cm^3^/year). The large variability of GM loss values across studies may be explained by differing scanner characteristics and sample sizes, the use of different image analysis protocols [29], and by different choices of covariates in the statistical models—for example, none, head size, age, and gender [30]. The present study combined the use of an automated image analysis method with a longitudinal design in a large community sample of older adults, thereby ensuring enhanced power and confidence in the estimation of the rate of GM atrophy.

We also performed cross-sectional analyses of our MRI data at baseline, which produced an age-related GM loss rate (−3.6 cm^3^/year) very close to that observed in the longitudinal analysis. The small difference between the cross-sectional and the longitudinal age-related effects can be attributed to the inherent differences in the estimation of these effects. Indeed, in the cross-sectional analysis, the age effect on GM volume is estimated over the complete age range of the study sample (17 years). The estimation of the regression slope between age and GM volume thus represent the loss of GM from 65 to 82 years (the age range of the study participants). This design is limited in its ability to account for large inter-individual variability in brain structure changes, and is restricted to studying the effect of chronological age on the brain [13]. In contrast, longitudinal GM rate of loss is directly computed for each participant, based on the difference between two measurements during the 4-year follow-up, and is thus able to address intra-individual effects of the process of aging on brain structure [15]. Another source of this discrepancy may be the differences in image processing between the longitudinal and cross-sectional approaches. As described in the methodological issue section, the registration of the second MRI image to the first one in the longitudinal analysis may create bias and overestimate the absolute value of the atrophy rate. However, since the two estimated rates were very similar, it is likely that this potential bias is small and that the real rate of GM atrophy measured in the present study lies in between −3.6 and −4.0 cm^3^/year.

The present results also showed that, after 65 years, the annualized global rate of GM atrophy remained constant with age, and that this stability was independent of sex. These findings represent a substantial contribution to the debate regarding the pattern of GM change in healthy older adults. Several cross-sectional studies have been performed in subjects spanning a large age range (14 to 80 years), and have demonstrated a marked non-linear pattern of maturation and age-related changes in most brain structures, and in the total GM [31]–[36]. However, these studies have included smaller samples of older adult participants than the present study, and thus do not allow a definitive conclusion on the pattern of age-related GM change in late life. Moreover, when using non-linear quadratic models, the inclusion of children and young adults in aging studies can affect the estimation of decline between 65 and 85 years of age [37]. The present cross-sectional and longitudinal findings indicated that a linear model can adequately model the relationship between age and GM volume among participants over 65 years of age, and that the annualized rate of GM atrophy stays constant over the age range of 65–85 years.

The present study also highlighted that, while men and women presented different educational level at entry time (men were more educated than women), there was no association between the education level and the rate of GM for either sex. The absence of education level on the rate of GM tissue loss argues in favor of the hypothesis stating that education might influence cognitive reserve through connectivity and/or synapses efficiency rather than by neuron numbers.

#### 1.2. Regional GM rate of atrophy

The presently estimated regional pattern of the rate of GM loss was very similar to that reported in previous longitudinal studies performed in older adults [4]–[5], [15]–[16]. This pattern involved GM loss over the entire cortex, with the highest rates in the frontal and parietal cortices, the hippocampus, the temporal pole, and the middle occipital gyri. Interestingly, the present analysis also revealed GM loss in the primary cortices (auditory, visual, and motor), with however a lower rate compared to that observed in multimodal associative regions, except for the heschl gyrus. The present results clearly demonstrated that the highest GM decline (by percentage) lies in the lateral and orbital parts of the prefrontal regions and in the parietal cortices. Across all age ranges, the prefrontal cortex is usually considered the structure most affected during normal aging [9], [38]–[39]. It is therefore a key region in the frontal aging theory, which states that a major component of cognitive aging is related to a structural deficit of the prefrontal cortex [40]. An investigation of 66 older adult participants with a baseline age range of 60–84 years showed that the rate of cortical thickness change measured over 8 years was higher in the frontal and parietal regions than that observed in the temporal and occipital lobes [16]. These results are in agreement with the pioneer work of Resnick et al., which demonstrated a similar pattern of longitudinal decline in GM volumes (frontal and parietal showed greater decline, compared with temporal and occipital lobar regions) [15]. In contrast, Fjell et al. reported that in 132 healthy older adults aged 75 years old, the rate of change of cortical thickness reduction measured over 2 years was higher in the temporal cortex (including the hippocampus and the amygdala) than in the frontal cortex [4]. In the same study, these authors reported hippocampus atrophy rates of −0.84% and −1.76% at 1-year and 2-year follow-ups, respectively [4]. In the present study, we found an atrophy rate of −1.05%/year measured over the 3.6-year follow-up period, slightly higher than the 0.88%/year of Fjell study. This difference can mainly by assigned to the different MRI analysis strategies (2D surface-based vs. 3D volumetric) between the two studies. The potential bias introduced by the present longitudinal analysis strategy (see the study limitation section), together with the partial volume effect in spatial normalization and the cerebral region segmentation procedure that we applied could partly explain the higher rate of hippocampus atrophy found here. However, another study investigating the rate of hippocampus atrophy in 42 older adult participants (aged over 58 years) over a similar period (3.5 years), applied a manual delineation procedure of this target structure, and found a rate of hippocampus volume loss very close to that in the present study (−1.0%/year) [14]. Finally, we further observed a lower rate of atrophy in the cerebellum, which is in accordance with previous studies that have revealed significant cerebellum shrinkage that increases from middle adulthood to old age [9]–[39].

### 2. The hippocampus: a specific site of acceleration of the rate of GM atrophy in late life

Although the global annual rate of GM atrophy was independent of age in our sample of healthy older adults, voxel-based analysis revealed the hippocampus to be a unique area where the loss of GM significantly accelerated with age after 65. The acceleration of hippocampus atrophy was found to be 35 times higher than the overall GM atrophy. Importantly, this acceleration was not due to a difference in the magnitudes of the rates of GM and hippocampus atrophy (−0.8%/year vs. −1.0%/year, respectively). Rather, this result demonstrated the enhanced vulnerability of the hippocampus as compared to other cerebral regions.

This finding is in agreement with results of previous longitudinal studies based on an *a priori* region-of-interest delineation approach. In a sample of participants aged over 58 years, Du et al. found that age was significantly associated with increased atrophy rates for the entorhinal cortex and the hippocampus [14]. Fjell et al. used an exploratory analysis without any *a priori* information to investigate 142 healthy older adults over 60 years of age, and reported significant correlations between age and one-year rates of atrophy in 32 ROIs, including the hippocampus, parahippocampus, entorhinal cortex, temporal, frontal, and parietal and occipital cortices [4]. Our findings are slightly different since we identified the hippocampus as the only site of acceleration of the rate of GM atrophy after 65 years of age (surviving the *p* <0.05 FWE corrected statistical threshold).

The apparent discrepancy between our findings and those of the Fjell et al. may be attributed to the different anatomical phenotypes studied (volume vs. cortical thickness, respectively). Indeed, it has been reported that cortical thickness is a less variable [41], more sensitive [42] and more reproducible [43] brain phenotype than grey matter volume. Thus, it is possible that surface-based analysis could be more sensitive, revealing a larger set of cortical areas in which age correlates with the atrophy rate. However, Fjell et al. emphasized that the occurrence of preclinical Alzheimer's disease is much higher among 70- and 80-year-olds than among 50- and 60-year-olds [4]. It is thus also possible that the pattern of accelerated longitudinal atrophy with age in healthy older adults could be mostly related to preclinical Alzheimer's disease in a subgroup of participants, even though cognitive symptoms have not yet manifested. Therefore, potential difference in the proportion of preclinical Alzheimer's disease participants between the Fjell et al. study and the present study may partly explain the amount of correlation between age and cortical rate of loss. Regardless of these differences, our present findings emphasize the specific vulnerability of the hippocampus to the aging process, making age a key factor of hippocampus atrophy.

### 3. Sex effects on the rate of GM atrophy

In the present study we demonstrated a significant sex difference in the dynamics of brain aging patterns in healthy older adults by revealing that after 65 years of age, women experienced larger rates of GM atrophy than men. This result is in line with our previous findings in a different cross-sectional cohort of healthy older adults, namely the EVA-MRI. In this previous study, 662 participants over 60 years of age showed a trend of a higher rate of GM atrophy in women (−2.7 cm^3^/year) compared to men (−1.7 cm^3^/year) [6]. Interestingly, the previously observed difference between the sex-related rates of GM atrophy (−1.0 cm^3^/year) was similar to that found in the present study (−1.4 cm^3^/year). The significance of the difference in the present study and not in the former is likely due to both the difference in sample sizes (662 vs. 1,172) and the increased statistical power of the reduced variance of the longitudinal design.

**Figure 7:**
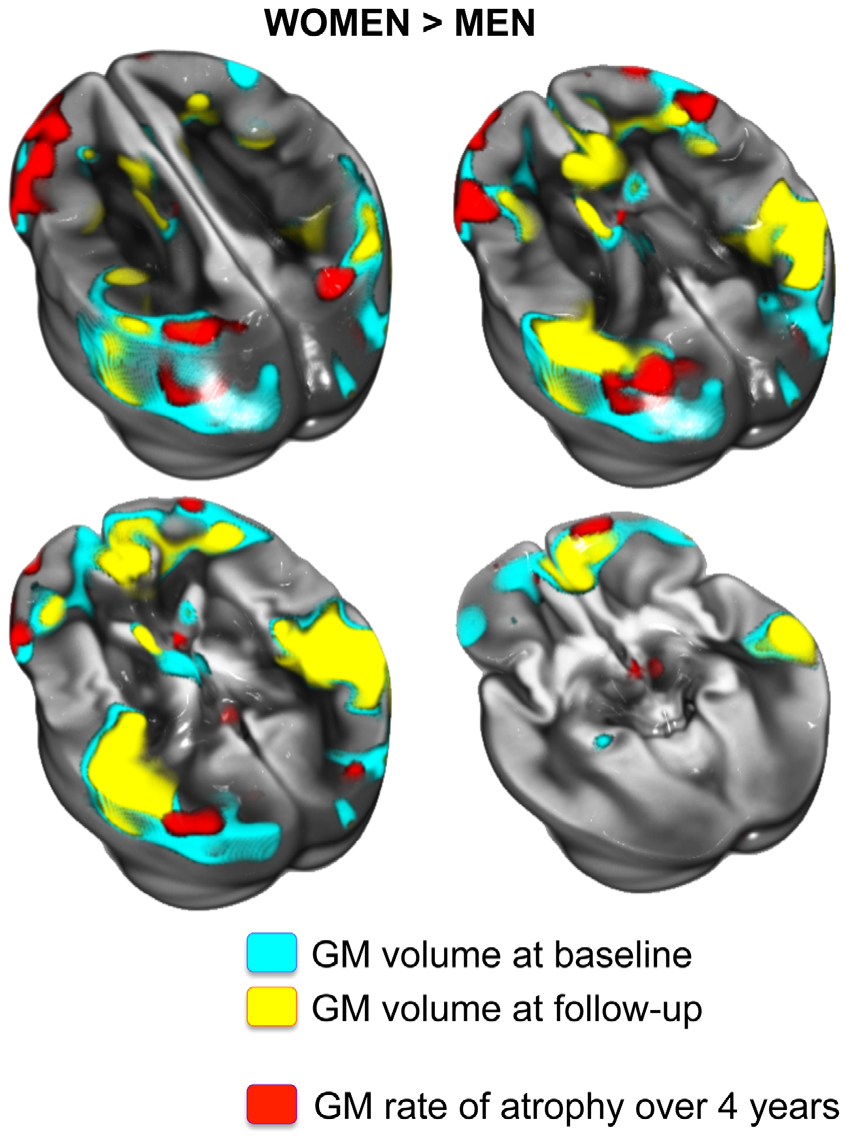
Cortical areas in which women presented a higher GM volume than men at baseline (in blue) and at follow-up (in yellow). Areas where women had a higher rate of atrophy than men are presented in red. These regional sex-related differences are displayed at a significance threshold of *p*<0.05 (FWE corrected) and superimposed onto the mean GM map of the 3C-MRI cohort (*n* = 1,172).

Here we found that compared to men, women presented a higher rate of GM atrophy specifically in the inferior frontal gyri, the inferior and superior parietal gyri, and the left middle occipital gyrus (see Figure 5). It is noticeable that this regional pattern of sex-related differences in rate of GM atrophy largely overlaps the set of areas where women presented a higher regional GM volume at baseline (see Figure 7). Four years later, women still presented higher regional GM volumes than men in almost the same set of areas, although the spatial extent of larger GM volume areas in women was considerably reduced mainly due to the regional sex-related effect on the GM rate of atrophy in these same regions. This observation raises the question of whether the difference in baseline GM volume may cause the higher rate of GM atrophy in these same regions. To test this hypothesis, we performed the same analysis including the baseline GM volume as a covariate (instead of the total intracranial volume), and observed the same pattern of regions for which women present a higher GM rate of atrophy. These results suggest that the higher regional GM rate of atrophy observed in women was not due to the greater amount of GM at baseline.

Our present results differed from those of a previous report that showed a greater decline in cortical thickness in men relative to women in several brain regions, including middle frontal, inferior parietal, superior parietal, postcentral, parahippocampal, precuneus, cingulate, and superior temporal gyri [16]. van Velsen et al. also found a more apparent decrease of cortical thickness in the frontal lobe in 488 men compared to in 534 women in a large cross-sectional study [8]. Understanding this sexual dimorphism in rate of GM loss requires knowledge about sex-related cerebral differences that may have occurred before the age of 65 years. It is possible that the temporal dynamic of GM atrophy between men and women differs across the lifespan. The differences observed in regional GM volume at baseline between women and men (see Figure 7) emphasize the idea that men had reduced GM volume in these areas at the age of 70, probably due to a higher rate of GM atrophy before their seventh decade. A protective effect of female hormones is one potential cause of early GM atrophy in men, or of delayed GM atrophy in women [44]–[46]. We were able to test this hypothesis in our present study, and showed that the HRT+ women presented a significant lower annualized rate of GM atrophy than HRT− women. This result is in agreement with the more pronounced age-related effect on cerebral tissue that has already been reported in HRT− women [47]–[50] due to the non-compensated decrease in estrogen level at menopause [51]. This last finding, together with the low frequency of HRT+ women in the 3C-MRI cohort, may at least partly explain the observed sex difference in rates of GM atrophy among older adults.

### 4. Methodological issues

#### 4.1. Baseline and follow-up image registration

Analysis of longitudinal MRI data is very challenging, and it is quite easy to introduce bias in the processing pipeline. One limitation of the present study can come from the fact that the T1-weighted follow-up image was aligned to the baseline T1-weighted image thus producing a different smoothness of data only for the second time-point co-registered to the first one. In this case, the baseline scan is used as a reference frame and therefore is treated differently from the other time-point. This interpolation asymmetry can introduce a potential bias in the estimation of the rate of change: generally an overestimation [52]–[54]. In order to limit this type of effect, it is thus preferable to treat all time points identically and ensure they undergo the same degree of smoothing due to image interpolation by creating an unbiased within-subject template space for the registration of all images of a given subjects [55]. Despite this source of bias, it is worth noticing that GM volume changes measured longitudinally were only 10% higher that GM volume decrease using cross-sectional data at baseline. Additionally, the potential bias on the rate of GM atrophy we measured is unlikely to impact the main results of the present study, since this bias has no reason to be dependent on the sex or the age of the subjects.

Another limitation of the present study is the relatively low degree of freedom of the spatial registration algorithms used to align the baseline and follow-up to the template reference space. Use of more accurate spatial normalization algorithm (for example the diffeomorphic DARTEL software [56], [57] would have probably reduced the residual inter-subject variability after normalization and therefore the GM volume and rate of change estimation, but would not have changed the main outcomes of our study. However the relatively low resolution and contrast of the 1.5T T1 MRI acquisitions available in the present study limits the use of very high degree of freedom normalization procedures available in the more recent versions of the SPM package, nor the use of more sophisticated brain anatomical phenotype measurements such as cortical thickness. Note that the robustness of the procedure analysis use in the present study has been previously validated [6], [23], [58]–[59].

#### 4.2. Hippocampus measurement

Another possible limitation of the present study can come from the way we estimated the hippocampal volume, which was not based on individual definition but relied on the AAL atlas hippocampus ROI. Note that the latter approach has been previously validated, providing results in accordance with non *a priori* VBM statistical analysis [23]. Nevertheless, it is still possible that residual inter-subject variability after normalization could bias hippocampal volume estimation through partial volume effects due to adjacent structures. However, several arguments can be used to discard this hypothesis. Firstly, our approach provided an average total hippocampus volume of 6.68±0.83 cm^3^ at baseline in agreement with values reported in numerous MRI studies. For example, den Heijer et al. reported, using manual segmentation, a total hippocampal volume of 6.4 cm^3^ measured on a sample of 949 healthy elderly subjects [60]. Secondly, although one must expect the variability of our hippocampal volume estimates to heavily rely on the accuracy of the spatial normalization process, we anticipated the latter to be good since it has been previously demonstrated that residual anatomical variability between subjects after spatial normalization is lowest for internal structures such as the hippocampus in which average overlap in left and right hippocampus reaches 60%, to be compared to a 35% overlap in the entire GM [61]. As a matter of fact, hippocampal volume coefficient of variation in our sample was found to be close to 10%, a value similar to what was reported in the den Heijer et al. study [60]. Although a potential bias in hippocampal volume estimation is still present, it's important to recall that manual segmentation would be infeasible for such a large dataset, and that the implementation of more accurate volume estimates developed in recent years would be of great interest [62].

#### 4.3. Laterality effect on rate of GM atrophy

Figure 1 and Figure 5A illustrate a potential laterality effect on GM rate of atrophy that was not investigated in the present study since the methodological design used here did not allow us to statistically test for differences between the left and right hemispheres. Indeed, the 3C-MRI template used for spatial normalization was not a symmetrical one, and use of asymmetrical templates for both spatial normalization and segmentation is known to introduce biases in the study of hemispheric anatomical features [63]. Moreover the AAL parcellation used to estimate the regional GM rate of atrophy is also asymmetric. As a consequence, hemispheric GM volumes and evolution of these volumes during the 4 years follow-up would also be biased. This is precisely why we chose to pool the left and right hemispheric region of the AAL atlas to investigate the regional quantification of the rate of GM atrophy. Furthermore, in order to understand the hemispheric effect of brain aging in older participants we first need to precisely describe the GM asymmetry pattern in these participants and this entire topic requires additional observation to be fully addressed.

## Conclusions

The present study reports a global and regional description of the annualized rate of grey matter loss and its evolution after the age of 65, in a 4-year longitudinal community cohort performed in 1,172 healthy older participants. We highlighted that the global annualized rate of grey matter atrophy remained constant throughout the age range of the cohort, in both sexes. This pattern was also replicated at the regional level, with the striking exception of the bilateral hippocampus, which showed a rate of grey matter atrophy that significantly accelerated with age, emphasizing a specific vulnerability of the hippocampus to the aging processes after 65 years of age. Moreover, the rate of grey matter atrophy was found higher in women than men, suggesting a greater anatomical vulnerability of women in late life.

## Supporting Information

Table S1 **Raw data of the study sample.**
(XLSX)

## References

[1] Raz N, Rodrigue KM (2006) Differential aging of the brain: patterns, cognitive correlates and modifiers. Neurosci Biobehav Rev 30:730–748. 16919333 10.1016/j.neubiorev.2006.07.001 PMC6601348

[2] Klöppel S, Stonnington CM, Chu C, Draganski B, Scahill RI, et al (2008) Automatic classification of MR scans in Alzheimer's disease. Brain 131:681–689. 18202106 10.1093/brain/awm319 PMC2579744

[3] Lerch JP, Pruessner J, Zijdenbos AP, Collins DL, Teipel SJ, et al (2008) Automated cortical thickness measurements from MRI can accurately separate Alzheimer's patients from normal elderly controls. Neurobiol Aging 29:23–30. 17097767 10.1016/j.neurobiolaging.2006.09.013

[4] Fjell AM, Walhovd KB, Fennema-Notestine C, McEvoy LK, Hagler DJ, et al (2009) One-year brain atrophy evident in healthy aging. J Neurosci 29:15223–15231. 19955375 10.1523/JNEUROSCI.3252-09.2009 PMC2827793

[5] Driscoll I, Davatzikos C, An Y, Wu X, Shen D, et al (2009) Longitudinal pattern of regional brain volume change differentiates normal aging from MCI. Neurology 72:1906–1913. 19487648 10.1212/WNL.0b013e3181a82634 PMC2690968

[6] Lemaître H, Crivello F, Grassiot B, Alpérovitch A, Tzourio C, et al (2005) Age- and sex-related effects on the neuroanatomy of healthy elderly. Neuroimage 26:900–911. 15955500 10.1016/j.neuroimage.2005.02.042

[7] Ikram MA, Vrooman HA, Vernooij MW, van der Lijn F, Hofman A, et al (2008) Brain tissue volumes in the general elderly population. The Rotterdam Scan Study. Neurobiol Aging 29:882–890. 17239994 10.1016/j.neurobiolaging.2006.12.012

[8] van Velsen EF, Vernooij MW, Vrooman HA, van der Lugt A, Breteler MM, et al (2013) Brain cortical thickness in the general elderly population: The Rotterdam Scan Study. Neurosci Lett 550:189–194. 23831346 10.1016/j.neulet.2013.06.063

[9] Raz N, Ghisletta P, Rodrigue KM, Kennedy KM, Lindenberger U (2010) Trajectories of brain aging in middle-aged and older adults: regional and individual differences. Neuroimage 51:501–511. 20298790 10.1016/j.neuroimage.2010.03.020 PMC2879584

[10] Fox NC, Ridgway GR, Schott JM (2011) Algorithms, atrophy and Alzheimer's disease: cautionary tales for clinical trials. Neuroimage 57:15–18. 21296168 10.1016/j.neuroimage.2011.01.077

[11] Davatzikos C, Xu F, An Y, Fan Y, Resnick SM (2009) Longitudinal progression of Alzheimer's-like patterns of atrophy in normal older adults: the SPARE-AD index. Brain 132:2026–2035. 19416949 10.1093/brain/awp091 PMC2714059

[12] Clark VH, Resnick SM, Doshi J, Beason-Held LL, Zhou Y, et al (2012) Longitudinal imaging pattern analysis (SPARE-CD index) detects early structural and functional changes before cognitive decline in healthy older adults. Neurobiol Aging 33:2733–2745. 22365049 10.1016/j.neurobiolaging.2012.01.010 PMC4023476

[13] Fjell AM, Westlye LT, Grydeland H, Amlien I, Espeseth T, et al (2014) Accelerating Cortical Thinning: Unique to Dementia or Universal in Aging? Cereb Cortex 24:919–934. 23236213 10.1093/cercor/bhs379 PMC3948495

[14] Du AT, Schuff N, Chao LL, Kornak J, Jagust WJ, et al (2006) Age effects on atrophy rates of entorhinal cortex and hippocampus. Neurobiol Aging 27:733–740. 15961190 10.1016/j.neurobiolaging.2005.03.021 PMC1779763

[15] Resnick SM, Pham DL, Kraut MA, Zonderman AB, Davatzikos C (2003) Longitudinal magnetic resonance imaging studies of older adults: a shrinking brain. J Neurosci 23:3295–3301. 12716936 10.1523/JNEUROSCI.23-08-03295.2003 PMC6742337

[16] Thambisetty M, Wan J, Carass A, An Y, Prince JL, et al (2010) Longitudinal changes in cortical thickness associated with normal aging. Neuroimage 52:1215–1223. 20441796 10.1016/j.neuroimage.2010.04.258 PMC2910226

[17] Fotenos AF, Snyder AZ, Girton LE, Morris JC, Buckner RL (2005) Normative estimates of cross-sectional and longitudinal brain volume decline in aging and AD. Neurology 64:1032–1039. 15781822 10.1212/01.WNL.0000154530.72969.11

[18] Du AT, Schuff N, Zhu XP, Jagust WJ, Miller BL, et al (2003) Atrophy rates of entorhinal cortex in AD and normal aging. Neurology 60:481–486. 12578931 10.1212/01.wnl.0000044400.11317.ec PMC1851672

[19] Alpérovitch A, Amouyel P, Dartigues J, Ducimetiere P, Mazoyer B, et al (2002) Epidemiological studies on aging in France: from the PAQUM1 study to the Three-City study. C R Biol 325:665–672. 12360853 10.1016/s1631-0691(02)01476-2

[20] Folstein M, Folstein S, McHugh P (1975) “Mini-mental state”. A practical method for grading the cognitive state of patients for the clinician. J Psychiatr Res 12:189–198. 1202204 10.1016/0022-3956(75)90026-6

[21] Woods R, Cherry S, Mazziotta J (1992) Rapid automated algorithm for aligning and reslicing PET images. J Comput Assist Tomogr. 16:620–633. 1629424 10.1097/00004728-199207000-00024

[22] Good CD, Johnsrude IS, Ashburner J, Henson RN, Friston KJ, et al (2001) A voxel-based morphometric study of ageing in 465 normal adult human brains. Neuroimage 14:21–36. 11525331 10.1006/nimg.2001.0786

[23] Lemaître H, Crivello F, Dufouil C, Grassiot B, Tzourio C, et al (2005) No epsilon4 gene dose effect on hippocampal atrophy in a large MRI database of healthy elderly subjects. Neuroimage 24:1205–1213. 15670698 10.1016/j.neuroimage.2004.10.016

[24] Tzourio-Mazoyer N, Landeau B, Papathanassiou D, Crivello F, Etard O, et al (2002) Automated anatomical labeling of activations in SPM using a macroscopic anatomical parcellation of the MNI MRI single-subject brain. Neuroimage 15:273–289. 11771995 10.1006/nimg.2001.0978

[25] Resnick SM, Goldszal AF, Davatzikos C, Golski S, Kraut MA, et al (2000) One-year age changes in MRI brain volumes in older adults. Cereb Cortex 10:464–472. 10847596 10.1093/cercor/10.5.464

[26] study of brain morphometrics using quantitative magnetic resonance imaging and difference image analysis. Neuroimage 20:22–33. 14527567 10.1016/s1053-8119(03)00219-2

[27] Smith C, Chebrolu H, Wekstein D, Schmitt F, Markesbery W (2007) Age and gender effects on human brain anatomy: A voxel-based morphometric study in healthy elderly. Neurobiol Aging 28:1075–1087. 16774798 10.1016/j.neurobiolaging.2006.05.018

[28] Greenberg DL, Messer DF, Payne ME, Macfall JR, Provenzale JM, et al (2008) Aging, gender, and the elderly adult brain: an examination of analytical strategies. Neurobiol Aging 29:290–302. 17049410 10.1016/j.neurobiolaging.2006.09.016 PMC2694568

[29] Pell GS, Briellmann RS, Chan CH, Pardoe H, Abbott DF, et al (2008) Selection of the control group for VBM analysis: influence of covariates, matching and sample size. Neuroimage 41:1324–1335. 18467131 10.1016/j.neuroimage.2008.02.050

[30] Barnes J, Ridgway GR, Bartlett J, Henley SM, Lehmann M, et al (2010) Head size, age and gender adjustment in MRI studies: a necessary nuisance? Neuroimage 53:1244–1255. 20600995 10.1016/j.neuroimage.2010.06.025

[31] Allen JS, Bruss J, Brown CK, Damasio H (2005) Normal neuroanatomical variation due to age: the major lobes and a parcellation of the temporal region. Neurobiol Aging 26:1245–60. 16046030 10.1016/j.neurobiolaging.2005.05.023

[32] Walhovd KB, Fjell AM, Reinvang I, Lundervold A, Dale AM, et al (2005) Effects of age on volumes of cortex, white matter and subcortical structures. Neurobiol Aging 26:1261–70. 16005549 10.1016/j.neurobiolaging.2005.05.020

[33] Kennedy KM, Erickson KI, Rodrigue KM, Voss MW, Colcombe SJ, et al (2009) Age-related differences in regional brain volumes: a comparison of optimized voxel-based morphometry to manual volumetry. Neurobiol Aging 30:1657–1676. 18276037 10.1016/j.neurobiolaging.2007.12.020 PMC2756236

[34] Walhovd KB, Westlye LT, Amlien I, Espeseth T, Reinvang I, et al (2011) Consistent neuroanatomical age-related volume differences across multiple samples. Neurobiol Aging 32:916–932. 19570593 10.1016/j.neurobiolaging.2009.05.013 PMC4040218

[35] Taki Y, Thyreau B, Kinomura S, Sato K, Goto R, et al (2011) Correlations among brain gray matter volumes, age, gender, and hemisphere in healthy individuals. PLoS One 6:e22734. 21818377 10.1371/journal.pone.0022734 PMC3144937

[36] Fjell AM, Westlye LT, Grydeland H, Amlien I, Espeseth T, et al (2013) Critical ages in the life course of the adult brain: nonlinear subcortical aging. Neurobiol Aging 34:2239–2247. 23643484 10.1016/j.neurobiolaging.2013.04.006 PMC3706494

[37] Fjell AM, Walhovd KB, Westlye LT, Østby Y, Tamnes CK, et al (2010) When does brain aging accelerate? Dangers of quadratic fits in cross-sectional studies. Neuroimage 50:1376–1383. 20109562 10.1016/j.neuroimage.2010.01.061

[38] Jernigan TL, Archibald SL, Fennema-Notestine C, Gamst AC, Stout JC, et al (2001) Effects of age on tissues and regions of the cerebrum and cerebellum. Neurobiol Aging 22:581–594. 11445259 10.1016/s0197-4580(01)00217-2

[39] Raz N, Lindenberger U, Rodrigue K, Kennedy K, Head D, et al (2005) Regional brain changes in aging healthy adults: general trends, individual differences and modifiers. Cereb Cortex 15:1676–1689. 15703252 10.1093/cercor/bhi044

[40] West R (1996) An Application of Prefrontal Cortex Function Theory to Cognitive Aging. Psychol Bull 120:272–292. 8831298 10.1037/0033-2909.120.2.272

[41] Hutton C, Draganski B, Ashburner J, Weiskopf N (2009) A comparison between voxel-based cortical thickness and voxel-based morphometry in normal aging. Neuroimage 48:371–380. 19559801 10.1016/j.neuroimage.2009.06.043 PMC2741580

[42] Burggren AC, Zeineh MM, Ekstrom AD, Braskie MN, Thompson PM, et al (2008) Reduced cortical thickness in hippocampal subregions among cognitively normal apolipoprotein E ε4 carriers. Neuroimage 41:1177–1183. 18486492 10.1016/j.neuroimage.2008.03.039 PMC2601686

[43] Dickerson B, Fenstermacher E, Salat D, Wolk D, Maguire R, et al (2008) Detection of cortical thickness correlates of cognitive performance: Reliability across MRI scan sessions, scanners, and field strengths. Neuroimage 39:10–18. 17942325 10.1016/j.neuroimage.2007.08.042 PMC2141650

[44] Eberling JL, Wu C, Haan MN, Mungas D, Buonocore M, et al (2003) Preliminary evidence that estrogen protects against age-related hippocampal atrophy. Neurobiol Aging 24:725–732. 12885580 10.1016/s0197-4580(02)00056-8

[45] Resnick SM, Espeland MA, Jaramillo SA, Hirsch C, Stefanick ML, et al (2009) Postmenopausal hormone therapy and regional brain volumes: the WHIMS-MRI Study. Neurology 72:135–142. 19139364 10.1212/01.wnl.0000339037.76336.cf PMC2677493

[46] Goto M, Abe O, Miyati T, Inano S, Hayashi N, et al (2011) 3 Tesla MRI detects accelerated hippocampal volume reduction in postmenopausal women. J Magn Reson Imaging 33:48–53. 21182120 10.1002/jmri.22328

[47] Cook IA, Morgan ML, Dunkin JJ, David S, Witte E, et al (2002) Estrogen replacement therapy is associated with less progression of subclinical structural brain disease in normal elderly women: a pilot study. Int J Geriatr Psychiatry 17:610–618. 12112157 10.1002/gps.644

[48] Raz N, Rodrigue KM, Kennedy KM, Acker JD (2004) Hormone replacement therapy and age-related brain shrinkage: regional effects. Neuroreport 15:2531–2534. 15538189 10.1097/00001756-200411150-00020

[49] Erickson KI, Colcombe SJ, Raz N, Korol DL, Scalf P, et al (2005) Selective sparing of brain tissue in postmenopausal women receiving hormone replacement therapy. Neurobiol Aging 26:1205–1213. 15917105 10.1016/j.neurobiolaging.2004.11.009

[50] Robertson D, Craig M, van Amelsvoort T, Daly E, Moore C, et al (2009) Effects of estrogen therapy on age-related differences in gray matter concentration. Climacteric 12:301–309. 19415541 10.1080/13697130902730742

[51] Gandy S (2003) Estrogen and neurodegeneration. Neurochem Res 28:1003–1008. 12737524 10.1023/a:1023246921127

[52] Thomas AG, Marrett S, Saad ZS, Ruff DA, Martin A, et al (2009) Functional but not structural changes associated with learning: an exploration of longitudinal voxel-based morphometry (VBM). Neuroimage 48:117–125. 19520171 10.1016/j.neuroimage.2009.05.097 PMC2981435

[53] Yushkevich PA, Avants BB, Das SR, Pluta J, Altinay M, et al (2010) Bias in estimation of hippocampal atrophy using deformation-based morphometry arises from asymmetric global normalization: an illustration in ADNI 3 T MRI data. Neuroimage 50:434–445. 20005963 10.1016/j.neuroimage.2009.12.007 PMC2823935

[54] Thompson W, Holland D (2011) Bias in tensor based morphometry Stat-ROI measures may result in unrealistic power estimates. Neuroimage 57:1–4. 21349340 10.1016/j.neuroimage.2010.11.092 PMC3471806

[55] Reuter M, Fischl B (2011) Avoiding asymmetry-induced bias in longitudinal image processing. Neuroimage 57:19–21. 21376812 10.1016/j.neuroimage.2011.02.076 PMC3260043

[56] Ashburner J, Friston K (2011) Diffeomorphic registration using geodesic shooting and Gauss–Newton optimisation. Neuroimage 55:954–967. 21216294 10.1016/j.neuroimage.2010.12.049 PMC3221052

[57] Ashburner J, Ridgway GR (2012) Symmetric diffeomorphic modeling of longitudinal structural MRI. Front Neurosci 6:197. 23386806 10.3389/fnins.2012.00197 PMC3564017

[58] Crivello F, Lemaître H, Dufouil C, Grassiot B, Delcroix N, et al**.** (2010) Effects of ApoE-epsilon4 allele load and age on the rates of grey matter and hippocampal volumes loss in a longitudinal cohort of 1186 healthy elderly persons. Neuroimage 53:, 1064–1069. 10.1016/j.neuroimage.2009.12.116 20060049

[59] Dumurgier J, Crivello F, Mazoyer B, Ahmed I, Tavernier B, et al**.** (2012) MRI atrophy of the caudate nucleus and slower walking speed in the elderly. Neuroimage 60, 871–878. 10.1016/j.neuroimage.2012.01.102 22305950

[60] den Heijer T, Oudkerk M, Launer LJ, van Duijn CM, Hofman A, et al (2002) Hippocampal, amygdalar, and global brain atrophy in different apolipoprotein E genotypes. Neurology 59:746–748. 12221169 10.1212/wnl.59.5.746

[61] Salmond H, Ashburner J, Vargha-Khadem F, Connelly A, Gadian G, et al (2002) The Precision of Anatomical Normalization in the Medial Temporal Lobe Using Spatial Basis Functions. Neuroimage 17:507–512. 12482103 10.1006/nimg.2002.1191

[62] Barnes J, Foster J, Boyes RG, Pepple T, Moore EK, et al (2008) A comparison of methods for the automated calculation of volumes and atrophy rates in the hippocampus. Neuroimage 40:1655–1671. 18353687 10.1016/j.neuroimage.2008.01.012

[63] Pepe A, Dinov I, Tohka J (2014) An automatic framework for quantitative validation of voxel based morphometry measures of anatomical brain asymmetry. Neuroimage 100:444–459. 24952229 10.1016/j.neuroimage.2014.06.029 PMC4457344

